# Effect of Parental Involvement on Children’s Academic Achievement in Chile

**DOI:** 10.3389/fpsyg.2019.01464

**Published:** 2019-06-27

**Authors:** Laura Lara, Mahia Saracostti

**Affiliations:** ^1^Department of Psychology, Universidad Autónoma de Chile, Talca, Chile; ^2^Centro de Investigación sobre Procesos Socioeducativos, Familias y Comunidades, Núcleo Científico Tecnológico en Ciencias Sociales y Humanidades, Universidad de La Frontera, Temuco, Chile

**Keywords:** parental involvement profiles, children’s academic achievement, elementary education, family and school relations, child development

## Abstract

Parental involvement in school has been demonstrated to be a key factor for children’s academic outcomes. However, there is a lack of research in Chile, as well as in Latin American countries in general, leaving a gap in the literature about the generalization of findings outside developed and industrialized countries, where most of the research has been done. The present study aims to analyse the associations between parental involvement in school and children’s academic achievement. Cluster analysis results from a sample of 498 parents or guardians whose children attended second and third grades in 16 public elementary schools in Chile suggested the existence of three different profiles of parental involvement (high, medium, and low) considering different forms of parental involvement (at home, at school and through the invitations made by the children, the teachers, and the school). Results show that there are differences in children’s academic achievement between the parental involvement profiles, indicating children whose parents have a low involvement have lower academic achievement. Findings are in line with international research evidence, suggesting the need to focus on this variable too in Latin American contexts.

## Introduction

On an international scale, parental involvement in school has long been heralded as an important and positive variable on children’s academic and socioemotional development. From an ecological framework, reciprocal positive interactions between these two key socializing spheres – families and schools – contribute positively to a child’s socioemotional and cognitive development (Bronfenbrenner, 1987). Empirical findings have demonstrated a positive association between parental involvement in education and academic achievement (Pérez Sánchez et al., 2013; Tárraga et al., 2017), improving children’s self-esteem and their academic performance (Garbacz et al., 2017) as well as school retention and attendance (Ross, 2016). Family involvement has also been found to be associated with positive school attachment on the part of children (Alcalay et al., 2005) as well as positive school climates (Cowan et al., 2012). Research has also evidenced that programs focused on increasing parental involvement in education have positive impacts on children, families, and school communities (Jeynes, 2012; Catalano and Catalano, 2014).

Parent-school partnership allows for the conceptualization of roles and relationships and the impact on the development of children in a broader way (Christenson and Reschly, 2010). From this approach, families and schools are the main actors in the construction of their roles and forms of involvement, generating new and varied actions to relate to each other according to the specific educational context. The main findings in the family-school field show a positive influence of this partnership, contributing to academic achievement and performance, among other positive consequences (Epstein and Sander, 2000; Hotz and Pantano, 2015; Sebastian et al., 2017).

There is also strong support from international research showing the positive influence of parental involvement over academic achievement, as has been demonstrated in a variety of meta-analyses across different populations and educational levels (Castro et al., 2015; Jeynes, 2016; Ma et al., 2016). Moreover, although there is a wide range of parental involvement definitions, some more general and others more specifics, there is a consensus among research results about the positive influence of parental involvement over child academic achievement. For example, in the meta-synthesis of Wilder (2014), where nine meta-analyses are analyzed, this influence was consistent throughout the studies, regardless the different definitions and measures used.

However, most of the studies on parental involvement in education hail from anglophone countries and are based on cross-sectional and correlational designs (Garbacz et al., 2017) while in Latin America research remains scarce. In a recent systematic review of the literature on parental involvement in education in Latin America, only one Mexican study from 1998 was found which was also heavily influenced by interventions from the United States (Roth Eichin and Volante Beach, 2018). Chile has acknowledged the importance of collaborative relationships between families and schools developing a National Policy for Fathers, Mothers and Legal Guardians Participation in the Educational System (Política de Participación de Padres, Madres y Apoderados/as en el Sistema Educativo) in 2002 which was recently updated in 2017 (Ministerio de Educación, Gobierno de Chile, 2017). Since the publication of this policy various local initiatives have sprouted in the country seeking to strengthen school family relations (Saracostti-Schwartzman, 2013). Nevertheless, the majority of research in the country has thus far been of a qualitative nature with a focus on describing relations between family members and their schools, and identifying tensions between these two spheres (Gubbins, 2011).

Thus, this study seeks to advance the analysis of the effects of parental involvement in school on the academic achievement of Chilean students. The study aims to analyse how different parental involvement profiles (based on the main forms of parental involvement identified in literature) influence children’s academic achieved. Parental involvement can take a wide variety of forms, among them, communication between family and school, supporting learning activities at home and involvement in school activities have been highlighted (Schueler et al., 2017), these are included in this study using the scales proposed by Hoover-Dempsey and Sandler (2005).

## Materials and Methods

### Participants and Procedure

The study included 498 parents or guardians whose children attended second and third grade in 16 public schools with high levels of socioeconomical vulnerability (over 85% according to official records of the schools) within three different regions in Chile (Libertador Bernando O’Higgins, Maule and Araucanía). Parents and guardians were aged between 20 and 89 years old (*M* = 35.02, SD = 7.02 for parents, *M* = 59.27, SD = 11.74 for grandparents and *M* = 43.14, SD = 15.41 for other guardians) and students between 7 and 12 (*M* = 8.30, SD = 0.93). The majority of them were mothers (83.9%). The majority of fathers and mothers had completed high school (33.1 and 40.6%, respectively), followed by elementary education (28.1 and 23.3%, respectively), no education completed (17.3% for both), professional title (7.2 and 6.8%, respectively) and university title (4.4 and 4.6%, respectively).

This study is part of a wider project focusing on the effectiveness of interventions aimed at strengthening the link between families and schools. This study has the approval of the Ethics Committee of the Universidad de La Frontera and the Chilean National Commission for Scientific and Technological Research (Acta 066-2017, Folio 036-17). Prior to data collection, after obtaining permission from the schools, informed consent forms were signed by the students’ legal guardians to authorize their participation. The data referring to the students (evaluation of learning outcomes) was compiled through official school records. The data referring to the families (parental involvement) was collected in paper format during parent teacher meetings at the end of the school year considering their behavior during the preceding year. Two research assistants trained for this purpose were present for the applications.

### Instruments

Parental involvement was assessed using the five scales proposed by Hoover-Dempsey and Sandler (2005) that aim to measure the level of family involvement in children’s education in elementary school from the point of view of the fathers, mothers and/or guardians. Scales have been adapted and validated by a panel of experts in Chile (Reininger, 2014). Scales included in this study are: (1) Parental involvement activities at home [five items, such as “*someone in this family (father, mother and/or guardian) helps the child study for test”* or “*someone in this family (father, mother and/or guardian) practices spelling, math or other skills with the child”*]; (2) Parental involvement activities at school (five items, such as *“someone in this family attends parent–teacher association meetings*” or “*someone in this family attends special events at school*”), (3) Child invitations for involvement (five items, such us “*my child asks me to talk with his or her teacher*” or “*my child asks me to supervise his or her homework*”); (4) Teacher invitations for involvement (six items, such as “*my child’s teacher asks me to help out at school*” or “*my child’s teacher asks me to talk with my child about the school day*”); and (5) General school invitations for involvement (six items, such as “*this school staff contact me promptly about any problem involving my child*” or “*parents’ activities are scheduled at this school so that we can attend*”). The first four scales have a four-point Likert response scale, that indicate the frequency of the items, from 0 (*never*) to 3 (*always*). The last scale has a 5-point Likert scale response, indicating the grade of agreement with the items, from 1 (*strongly disagree*) to 5 (*strongly agree*). Scales can be consulted as Supplementary Tables 1–5. Internal consistency of all scales was adequate (α = 0.79, α = 0.72, α = 0.72, α = 0.85, and α = 0.87, respectively).

Students’ academic achievement was evaluated thought the final average grade obtained at the end of the school year, recorded in a scale from 1 (*minimum achievement*) to 7 (*maximum achievement*).

## Results

**FIGURE 1:**
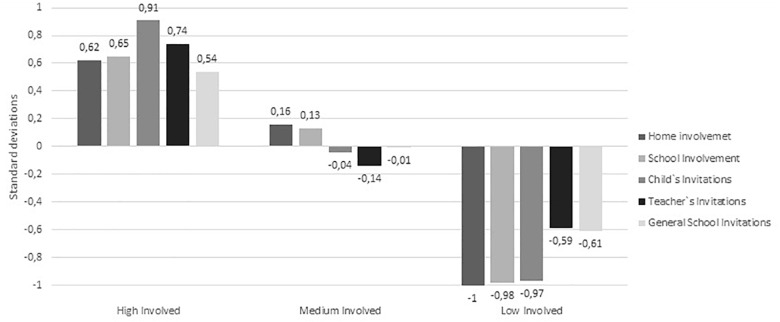
Parental involvement profiles.

Hierarchical cluster analysis was used to identify parental involvement profiles based on the five subscales of parental involvement scale (typified to avoid the influence of the different scale responses), applying the standardized Euclidian Distance method and using Ward’s algorithm. Cluster analyses results showed that the optimal solution was the grouping of the participants into three groups. In Figure 1 the typified scores of each of the variables considered to calculate the groups are shown.

To label the groups, we examined the family involvement profiles by computing a one-way ANOVA on the standardized scores of the five parental involvement scales with the clusters serving as the factors. The result revealed that the clustering variables significantly differed between the involvement scales [Parental involvement at home: *F*(2,497) = 147.83, *p* < 0.001, η2 = 0.37; Parental involvement at school: *F*(2,497) = 148.82, *p*< 0.001, η2 = 0.38; Child invitation for involvement: *F*(2,497) = 225.34, *p*< 0.001, η2 = 0.48; Teacher invitation for involvement: *F*(2,497) = 84.77, *p*< 0.001, η2 = 0.26; General school Invitation for involvement: *F*(2,497) = 53.38, *p*< 0.001, η2 = 0.18]. Scheffe *post hoc* multiple comparisons showed the differences were statistically significant between all the parental involvement profiles in all variables, with the first cluster scoring higher than the second and the third in all the scales, and the second higher that the third. Based on these differences and the scores, the first cluster was labeled as *High involved parents*, representing 144 parents (28.9%) that scored above the mean in all the involvement scales (from 0.54 to 0.91 standards deviations). The second cluster was named *Medium involved parent*s, including 228 parents (45.8%) that have scores close to the media in all the involvement scales (from -0.14 to 0.16 standards deviations). Finally, the third cluster was classified as *Low involved parents*, including 126 parents (25.3%) that scored below the mean in all the involvement scales (from -0.61 to -0.91 standards deviations). Table 1 shows demographic information for the clusters.

**Table 1:** Demographic information of the clusters.

	Cluster 1:	Cluster 2:	Cluster 3:
	High involved parents	Medium involved parents	Low involved parents
**Parent’s age**			
*M* (SD)	36.84 (9.72)	35.63 (8.42)	37.63 (10.28)
**Mother’s education %**			
No education completed	19.0	18.1	19.0
Elementary education completed	25.2	21.9	31.0
High school completed	43.0	44.8	43.1
Professional title	10.4	7.1	4.3
University title	2.2	8.1	2.6
**Father’s education %**			
No education completed	22.6	16.7	19.8
Elementary education completed	27.8	30.5	36.8
High school completed	36.1	38.6	34.0
Professional title	11.3	7.6	4.7
University title	2.3	6.7	4.7
**Child’s age**			
*M* (SD)	8.38 (0.98)	8.20 (0.86)	8.39 (0.97)
**Child’s %**			
Female	40.3	42.1	39.7
Male	59.7	57.9	60.3
**Child’s grade %**			
2°	43.8	50.9	49.2
3°	56.3	49.1	50.8

Finally, ANOVA results showed that there were significant differences in academic achievement scores between the three clusters of parent involvement profiles, *F*(2,430) = 5.37, *p* = 0.003, η2 = 0.03. Scheffe *post hoc* multiple comparisons showed that high (*M* = 5.97, SD = 0.49) and medium (*M* = 6.00, SD = 0.50) involved parents had children with higher academic achievement than low involved parents (*M* = 5.8, SD = 0.47). Complementarily, results from correlations between parental involvement and academic achievement scores support these results, showing a significant and positive correlation(*r* = 0.14, *p* = 0.003).

## Discussion

From the results presented, we can conclude the existence of three different profiles of parental involvement (high, medium and low) considering different scales of parental involvement (at home, at school and through the invitations made by the children, the teachers and the school). Secondly, results showed that there were differences in academic achievement scores between the parent involvement profiles, where high and medium involved parents had children with higher academic achievement than low involved parents.

As shown, international literature reveals that the degree of parental involvement is a critical element in the academic achievements of children, especially during their first school years highlighting the need to generate scientific evidence from the Chilean context. Most of the studies in this area come from anglophone countries (Garbacz et al., 2017) while in the Latin American context research is still scarce. Results from our study corroborate that parental involvement can contribute alike in other cultural contexts, pointing to the need to also implement policies to promote it.

In this context, Chile has acknowledged the importance of collaborative relationships between parents and schools leading to the development a National Policy for Father, Mother and Legal Guardian Participation. Nevertheless, most of the research in the country has thus far been of a qualitative nature with a focus on describing family-school relations and identifying tensions between these two spheres (Gubbins, 2011). Thus, this study seeks to make progress in the analysis of the effect of parental involvement and children’s and academic achievements of Chilean students.

## Ethics Statement

This study was carried out in accordance with the recommendations of the Chilean National Commission for Scientific and Technological Research with written informed consent from all subjects. All subjects gave written informed consent in accordance with the Declaration of Helsinki. The protocol was approved by the Ethics Committee of the Universidad de La Frontera and the Chilean National Commission for Scientific and Technological Research.

## Author Contributions

MS developed the study concept and the study design. LL substantially contributed to the study concept, and performed the data analysis and interpretation. MS and LL drafted the manuscript. All the authors approved the final version of the manuscript. They also agreed to be accountable for all aspects of the work.

## Conflict of Interest Statement

The authors declare that the research was conducted in the absence of any commercial or financial relationships that could be construed as a potential conflict of interest.
