# Supplementary material for: Effect of Parental Involvement on Children’s Academic Achievement in Chile
Source: Front Psychol. 2019 Jun 27;10:1464. doi: 10.3389/fpsyg.2019.01464 (PMC6610476; doi:10.3389/fpsyg.2019.01464)
Supplement: Supplementary file 1 [file Table_1.DOCX]

**Supplementary material:** Scales used to measure parental involvement

Table 1. Parental involvement at home scale

| Items | Nunca *Never* | A veces  *Sometimes* | Frecuente  *Usually* | Siempre  *Always* |
| --- | --- | --- | --- | --- |
| Alguien en la familia (padre, madre y/o apoderados) habla con el niño(a) acerca del año escolar  *Someone in this family (father, mother and/or guardian) talks with the child about the school year* | 0 | 1 | 2 | 3 |
| Alguien en la familia (padre, madre y/o apoderados) supervisa las tareas del niño(a)  *Someone in this family (father, mother and/or guardian) supervises this child’s homework.* | 0 | 1 | 2 | 3 |
| Alguien en la familia (padre, madre y/o apoderados) ayuda al niño(a) a estudiar para un examen  *Someone in this family (father, mother and/or guardian) helps the child study for tests* | 0 | 1 | 2 | 3 |
| Alguien en la familia (padre, madre y/o apoderados) practica matemáticas, ortografía y otras materias con el estudiante  *Someone in this family (father, mother and/or guardian) practices spelling, math or other skills with the child* | 0 | 1 | 2 | 3 |
| Alguien en la familia (padre, madre y/o apoderados) lee con el niño(a)  *Someone in this family (father, mother and/or guardian) reads with this child* | 0 | 1 | 2 | 3 |

Table 2. Parental involvement at school scale

| Items | Nunca  *Never* | A veces  *Sometimes* | Frecuente  *Usually* | Siempre  *Always* |
| --- | --- | --- | --- | --- |
| Alguien en la familia (padre, madre y/o apoderados) colabora en la escuela  Someone in this family (father, mother and/or guardian) helps out at this child’s school | 0 | 1 | 2 | 3 |
| Alguien en la familia (padre, madre y/o apoderados) asiste a entrevista con el profesor en caso de ser citado  Someone in this family (father, mother and/or guardian) attends parent-teacher meeting when called in | 0 | 1 | 2 | 3 |
| Alguien en la familia (padre, madre y/o apoderados) acompaña al curso en paseos escolares  Someone in this family (father, mother and/or guardian) volunteers to go on class field trips | 0 | 1 | 2 | 3 |
| Alguien en la familia (padre, madre y/o apoderados) acude a las reuniones de apoderados  *Someone in this family (father, mother and/or guardian) attends parent-teacher association meetings* | 0 | 1 | 2 | 3 |
| Alguien en la familia (padre, madre y/o apoderados) asiste a las celebraciones organizadas por la escuela  Someone in this family (father, mother and/or guardian) attends special events at school | 0 | 1 | 2 | 3 |

Table 3. Child invitations for involvement scale

| Items | Nunca  *Never* | A veces  *Sometimes* | Frecuente  *Usually* | Siempre  *Always* |
| --- | --- | --- | --- | --- |
| Mi hijo(a) me pide ayuda cuando no entiende su tarea  *My child asks me for help when he/she doesn’t understand her/his homework* | 0 | 1 | 2 | 3 |
| Mi hijo(a) me pide que lo acompañe cuando hace sus tareas  My child asks me to supervise his or her homework | 0 | 1 | 2 | 3 |
| Mi hijo(a) me pide que asista a las celebraciones de la escuela  *My child asks me to attend special events at school* | 0 | 1 | 2 | 3 |
| Mi hijo(a) me pide que colabore en la escuela  *My child asks me to help out at school* | 0 | 1 | 2 | 3 |
| Mi hijo(a) me pide que hable con sus profesores  *My child asks me to talk with his or her teachers* | 0 | 1 | 2 | 3 |

Table 4. Teacher invitations for involvement scale

|  | Nunca  *Never* | A veces  *Sometimes* | Frecuente  *Usually* | Siempre  *Always* |
| --- | --- | --- | --- | --- |
| El profesor/a de mi hijo/a me pide que ayude a mi hijo(a) con las tareas  *My child’s teacher asks me to help my child with homework* | 0 | 1 | 2 | 3 |
| El profesor/a de mi hijo/a me pide que supervise a mi hijo(a) con las tareas  My child’s teacher asks me to supervise his or her homework | 0 | 1 | 2 | 3 |
| El profesor/a de mi hijo/a me pide que hable con mi hijo(a) acerca del día escolar  *My child’s teacher asks me to talk with my child about the school day* | 0 | 1 | 2 | 3 |
| El profesor/a de mi hijo/a me pide que asista a un evento especial en la escuela  *My child’s teacher asks me to help out at school events* | 0 | 1 | 2 | 3 |
| El profesor/a de mi hijo/a me pide que ayude en la escuela  My child’s teacher asks me to help out at the school | 0 | 1 | 2 | 3 |
| El profesor/a de mi hijo/a se comunica conmigo (por ejemplo envía notas, por teléfono o correo electrónico.)  *My child’s teacher contacts me (for example sends notes, phones or emails)* | 0 | 1 | 2 | 3 |

Table 5. General school invitations for involvement scale

|  | Muy en desacuerdo  *Strongly disagree* | En desacuerdo  *Disagree* | Ni en acuerdo ni en desacuerdo  *Neither in agreement or disagreement* | De acuerdo  Agree | Muy de acuerdo  *Strongly agree* |
| --- | --- | --- | --- | --- | --- |
| Los profesores en esta escuela se interesan y cooperan con el progreso de mi hijo(a)  *The teachers at this school are interested and cooperate with my child progress* | 1 | 2 | 3 | 4 | 5 |
| Me siento acogido (a) en esta escuela  *I feel accepted at this school* | 1 | 2 | 3 | 4 | 5 |
| Las actividades para padres y apoderados se llevan a cabo en la escuela para que podamos asistir  *Parents’ activities are scheduled at this school so that we can attend* | 1 | 2 | 3 | 4 | 5 |
| La escuela me informa acerca de eventos especiales y encuentros  *The school informs me about special events and meetings* | 1 | 2 | 3 | 4 | 5 |
| El personal de la escuela se comunica conmigo por cualquier problema con mi hijo(a)  *This school staff contact me promptly about any problem involving my child* | 1 | 2 | 3 | 4 | 5 |
| Los profesores de la escuela me mantienen informado(a) acerca del progreso académico de mi hijo(a)  *The teachers at this school keep me informed about my child’s academic progress* | 1 | 2 | 3 | 4 | 5 |
